# Anatomy of a hotspot: Cisplatin hotspots in the *tdk* gene of *Escherichia coli*

**DOI:** 10.1002/em.22635

**Published:** 2024-10-10

**Authors:** Courtney Young, Mackenzie Lee, Zoe Ge, Jeana Shin, Bella Bursulaya, Dana Sorensen, Arnav Saud, Ananya Sridharan, Ava Gonick, Nhu Phi, Kelly Nguyen, Shawal Bhalli, Jyotsna Hiranandani, Jeffrey H. Miller

**Affiliations:** ^1^ Department of Microbiology, Immunology, and Molecular Genetics, and the Molecular Biology Institute University of California, and the David Geffen School of Medicine Los Angeles California USA

**Keywords:** bacterial systems, cisplatin, mutagenesis

## Abstract

We previously reported that certain sub‐regions of the *thyA* gene of *Escherichia coli* are more mutable than others when many different mutagens and mutators are analyzed (Mashiach et al., *Mutation Research Fundamental Molecular Mechansims of Mutagenesis*, **821**: 111702, 2021). In this study, we focus on a single mutagen, cisplatin and verify that mutations occur preferentially at specific 3 bp sequences, but only when they appear in certain subregions of the gene. Moreover, we show that hotspots for some premutational lesions are camouflaged by the preferential repair effected by the *uvrA,B,C*‐encoded excision repair system, even when they appear on the same strand. We do this by using a novel reporter gene in *E. coli*, the *tdk* gene that codes for thymidine deoxykinase, and we describe some of the advantages of utilizing this detection system.

## 1 INTRODUCTION

Classic work by Seymour Benzer revealed that the distribution of mutations in a gene is not random (Benzer, 1961). Instead, mutations occur at favored sites, and when these sites are highly favored they are termed “hotspots.” This is true for both spontaneous mutations and those induced by mutagenic agents. In some cases hotspots are caused by special sequence configurations, such as a repeated set of base pairs, bp; for example, AAAAAA in the case of the Benzer hotspot in the rII gene of phage T4 (Pribnow et al., 1981), or CTGGCTGGCTGG in the case of *lacI* in *Escherichia coli*, (Farabaugh et al., 1978), as a “slipped mispairing” mechanism (Streisinger et al., 1966) results in more frequent loss or gain of one repeat unit. Other sequences signal enzymes to alter DNA in specific places that leads to hotspots, often with important implications. An example is the methylation of 5′‐CCAGG‐3′ sequences in *E. coli* at the second ‐C‐ on each strand to create 5‐methylcytosine that can deaminate to generate thymidine, thus making these sequences hotspots (Coulondre et al., 1978). Similarly, in mammalian cells the 5′‐C‐G‐3′ sequences are methylated at the ‐C‐ on each strand, again leading to increased mutation rates (e.g., Laird & Jaenisch, 1994). Another example is the activation‐induced cytosine deaminase that deaminates cytosines to uracil preferentially in specific trinucleotide motifs (Pham et al., 2003), mainly in the small region of the gene specifying the hyervariable regions of immunoglobulins, The resulting hotspots for mutation are one basis for antibody diversification. There are also special structures that can be involved in creating hotspots. Although secondary structures have been suggested as promoting more frequent mutations (Ripley & Glickman, 1983; Todd & Glickman, 1982), in only one case has this been convincingly demonstrated. Susan Lovett and coworkers showed that a strong hotspot occurs in a quasipalindrome in the *thyA* gene sequence in *E. coli* that results from a form of hairpin‐templated replicative misalignment (Viswanathan et al., 2000).

Despite all the work cited above, many base substitution hotspots are not easily explained. There are some known factors involved in making certain sites more favored by different mutagens than others. Clearly, the nature of the target for those mutagens that generate pre‐mutational lesions plays a role. For example, cisplatin (CPT), the subject of the work reported here, generates intrastrand crosslinks preferentially at 5′‐A‐G‐3′, and 5′‐G‐G‐3′, leading principally to A:T→T:A and G:C→T:A transversions at the 5′ purine (Burnouf et al., 1987, 1990, and references therein). Among the many potential sites for these pre‐mutational lesions, some are more favored. In the case of many mutagens and mutators the nearest neighbor sequence on both the 5′ and 3′ sides of the mutated base can play a role (Coulondre & Miller, 1977; Fernandez et al., 2020; Foster et al., 2018; Miller, 1985; Pienkowska et al., 1993). Thus, we can express the three‐base sequence as a “triplet,” with the mutated base in the middle, and the nearest neighbors on each end of the triplet. For CPT, the CAG and AGG triplets are among the most favored (Brouwer et al., 1981; de Boer and Glickman, 1989). There are 16 possible nearest neighbor combinations for each of the six possible base changes. This has led to extensive work with mammalian cell lines to generate mutational “signatures” for a large series of mutagens and carcinogens by whole genomic sequencing of treated cell lines (e.g., Phillips, 2018). However, even among mutational sites that involve the same type of base change with the same mutagen and that are part of the same triplet, there are wide variations of mutation frequencies that approach two orders of magnitude difference (see “Discussion” in Fernandez et al., 2020). The favored sites within a set of identical triplets represent the true hotspots. Thus in the *rpoB* gene of *E. coli*, there are two mutational sites within a CAG triplet on the same strand. After treatment with CPT, one site has 28 occurrences in one sample of independent mutants, and the second site has zero occurrences (Garibyan et al., 2003). There are numerous examples of strong hotspots within a set of sites that are part of identical triplets. Whole genome sequencing cannot reveal which identical triplets are true hotspots, because this requires recording multiple recurrences at the same site, and whole genome sequencing rarely, if ever, records a recurrence at the same site. Determining true hotspots is still the domain of single gene reporter systems.

We developed the *tdk* (thymidine deoxykinase) single‐gene knockout system as a reporter gene in *E. coli*. We first constructed a catalog of all the base substitution mutations that result in a TDK negative phenotype. (The full details of the complete system will be reported elsewhere.) In the work reported here, we use this system to examine the hotspots caused by CPT. Previously, we showed that some sub‐regions of the *thyA* gene are more mutable than others (Mashiach et al., 2021). Here, we show that a specific mutagen, CPT, has hotspots for certain triplets that cluster in a short region of the gene, but not when they appear in other regions of the gene. Also, we show that certain pre‐mutational lesions are preferentially repaired by the *uvrA,B,C*‐encoded excision repair system, not only in a strand specific manner, but also when they appear on the same strand. Thus, hotspots for certain premutational lesions are partially camouflaged by preferential repair.

## 2 MATERIALS AND METHODS

### 2.1 *E. coli* strains

One of two strains designated as wild‐type is CC107 (Cupples et al., 1990), which we have used in multiple mutagenesis studies (e.g., Garibyan et al., 2003; Miller et al., 2002). This strain background is: *ara ∆ (gpt‐lac)5 thi/F*′*128 lacIZ proA* ^+^ *B* ^+^. CC107 also carries a frameshift mutation in *lacZ* on the F′ plasmid. We have not observed any differences in the mutagenesis seen in this strain or in other wild‐type strains we have used (e.g., BW25113, Ang et al., 2016; see below BW25113, Datsenko & Wanner, 2000). The other wild‐type strain BW25113 is (*lacI* ^*q*^ *rrnB*_*T14*_ Δ*lacZ*_*WJ16*_ *hsdR514 ΔaraBAD*_*AH33*_ *ΔrhaBAD*_*LD78*_). This is the starting strain for the KEIO collection, described in Baba et al. (2006). The UvrA‐deficient strain used here is from the KEIO collection.

### 2.2 Media

The following media (Miller, 1992) were used. LB (10 g tryptone, 5 g yeast extract,10 g NaCl per liter). For plates, 15 g of agar was added per liter just prior to pouring. For mutant selections, LB medium was supplemented with 100 ng/mL AZT. AZT was prepared by first making a stock solution of 1 mg/mL in water, and then adding 100 μg to 1 L of sterile LB agar medium just prior to pouring plates. The stock solution was stored in a freezer and reused when needed.

### 2.3 Growth conditions, mutagenesis treatment

Unless otherwise stated, all genetic methods are as described by Miller (1972, 1992). CPT (cisplatin) treatment: Overnight cultures grown in a 37°C incubator were used to seed “overday” cultures by inoculating 250 μL into each of 5 separate 5 mL LB cultures that were then incubated in a 37°C water bath for 3–4 h. After placing on ice for 5 min, the cultures were pooled and spun down, the liquid discarded and the pellets washed in minimal A buffer (unsupplemented minimal A), recentrifuged and resuspended in half the initial LB volume in minimal A buffer. CPT was added to a final concentration of 100 μg/mL, and incubated for 60 min in a water bath at 37°C without shaking. (CPT was prepared in a 2 mg/mL stock solution in water that was shaken vigorously until dissolved, approximately 30 min. It was prepared fresh before each use). The cells were spun down and resuspended into 2.5 mL minimal A buffer. These cultures were titered for survival. Outgrowth cultures were seeded with 0.2 mL of this resuspension in 5 mL LB, grown overnight at 37°C on a rotor at 50 rpm (0.44 g) before plating. The treatments with CPT resulted in 46% survival, on average.

### 2.4 Determination of mutant frequencies

The cells grown as indicated above were plated on LB medium supplemented with 100 ng/mL AZT. Mutant frequency (f) was determined as the median frequency from a set of cultures (the number of cultures varied from 6 to 28), by dividing the number of AZT‐resistant mutants per ml by the titer of the culture as determined by plating on LB medium without AZT. 95% confidence limits were determined according to the method of Dixon and Massey Jr. (1969).

### 2.5 Chromosomal DNA isolation and sequencing

Chromosomal DNA was isolated from overnight cultures of each mutant after single colony purification by streaking. We only picked one AZT‐resistant mutant per culture. The PCR tests were carried out as colony PCR reactions. The *tdk* gene was PCR‐amplified from genomic DNA using Taq polymerase (Invitrogen) and sets of primers which allowed us to sequence directly from the PCR product. The PCR conditions were as follows. Annealing temperature 58°C, elongation time 1:11 min. The primer sequences were: tdk F (forward) 22mer 5′‐CAAGGCTTCGTAAGGGAGAACG‐3′, tdk R (reverse) 21mer 5′‐CTGCCGAGAAGGGTATATAGC‐3′; The sequencing primer was *tdk* F. The *tdk* F primer extends from 120 bases to 98 bases upstream of the 5′end of the gene. The reverse primer extends from 72 bases to 93 bases downstream of the 3′ end of the gene. The unpurified PCR product was outsourced to Laragen (Culver City) for purification with exoSap (Affymetrix) and Sanger sequencing.

### 2.6 Chemicals

CPT and AZT were purchased from Sigma (St. Louis, MO).

## 3 RESULTS

### 3.1 The tdk reporter gene system

**FIGURE 1:**
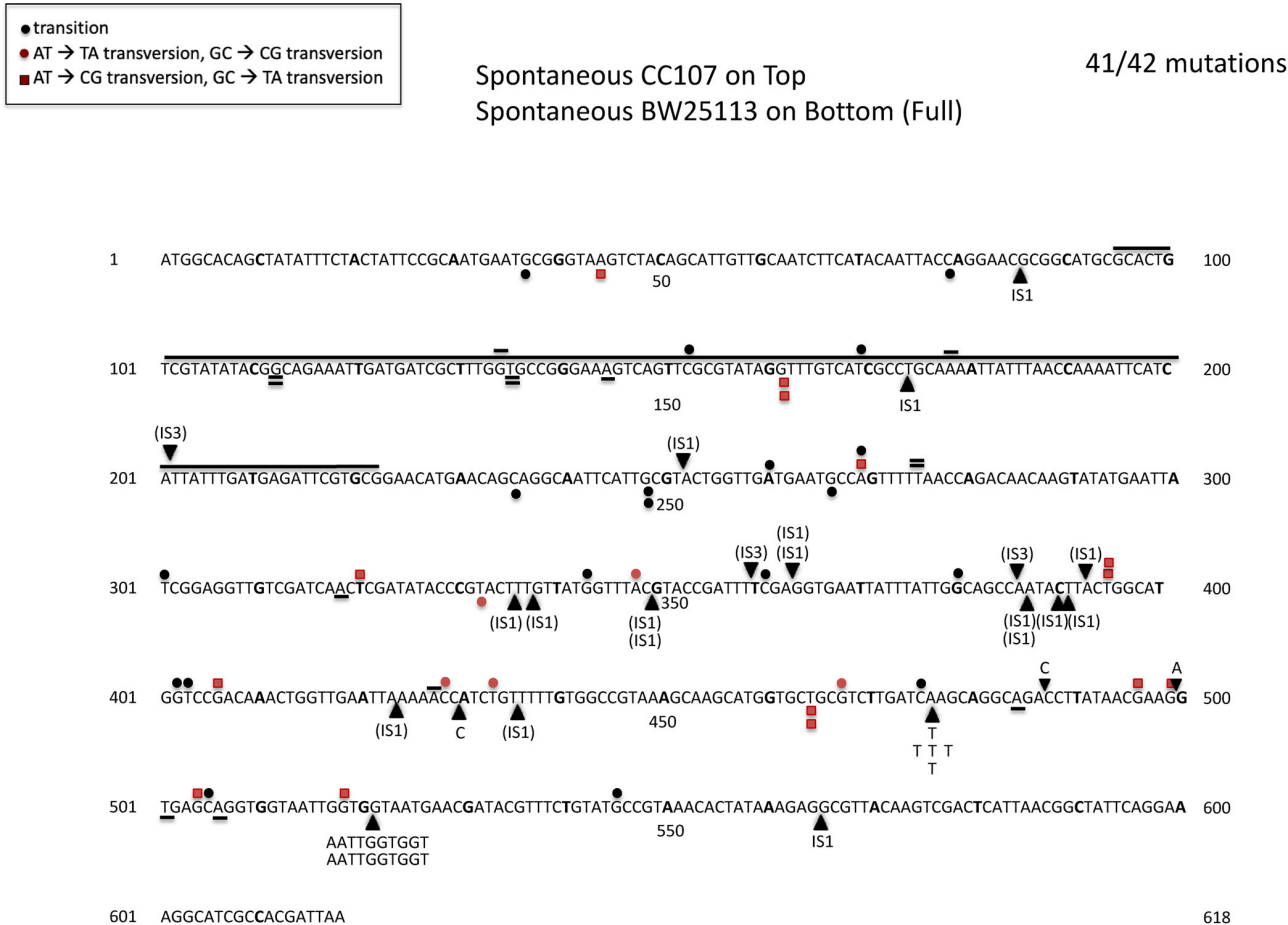
Distribution of spontaneous mutations in the *tdk* gene in CC107 and BW25113. The arrowheads indicate the point of insertions of either IS elements or single or multiple base pairs. Deletions are indicated by horizontal lines.

AZT (3′‐azido‐3′‐deoxythymidine) is a DNA replication chain blocker that is used in anti‐retroviral therapy (Ellwell et al., 1987; Furman et al., 1986; Olivero, 2007; Thompson & Lamont, 2019). The TDK protein is required to phosphorylate thymidine and thymdine derivatives such as 5‐fluorodeoxyuridine (Hiraga et al., 1967; Summers & Raksin, 1993). This is also true for AZT, as we find that a large majority of the mutants resistant to 100 ng/mL AZT have mutations that inactivate the *tdk*‐encoded thymidine deoxykinase. The advantages of using *tdk* as a reporter gene are that mutants grow overnight on LB plates with AZT, and the gene is only 618 bp long, meaning one can sequence mutations anywhere in the gene using a single primer pair. Also, all types of mutations can be detected, including insertions, deletions, and base substitutions. Moreover, the potency of AZT lasts several months for plates stored in a refrigerator. Figure 1 shows the spontaneous mutations detected in each of two closely related strain backgrounds, both of which have been used frequently to derive mutational spectra (e.g., Ang et al., 2016; Garibyan et al., 2003; Miller et al., 2002; Yaramada et al., 2022). The spontaneous background includes a large proportion of IS1 insertions. The base substitutions do not show prominent hotspots, making this an ideal system for analyzing base substitutions induced by mutagens or occurring in mutator strain backgrounds. We should note that the vast majority of the AZT‐resistant mutants display mutations in the *tdk* gene. Typically, 1%–5% of the sequenced mutants do not show a mutation in *tdk*. We cannot tell at this stage whether that reflects another small locus being involved. In any case, the proportion is small enough to not interfere with the utility of this system.

### 3.2 CPT induced mutations

**FIGURE 2:**
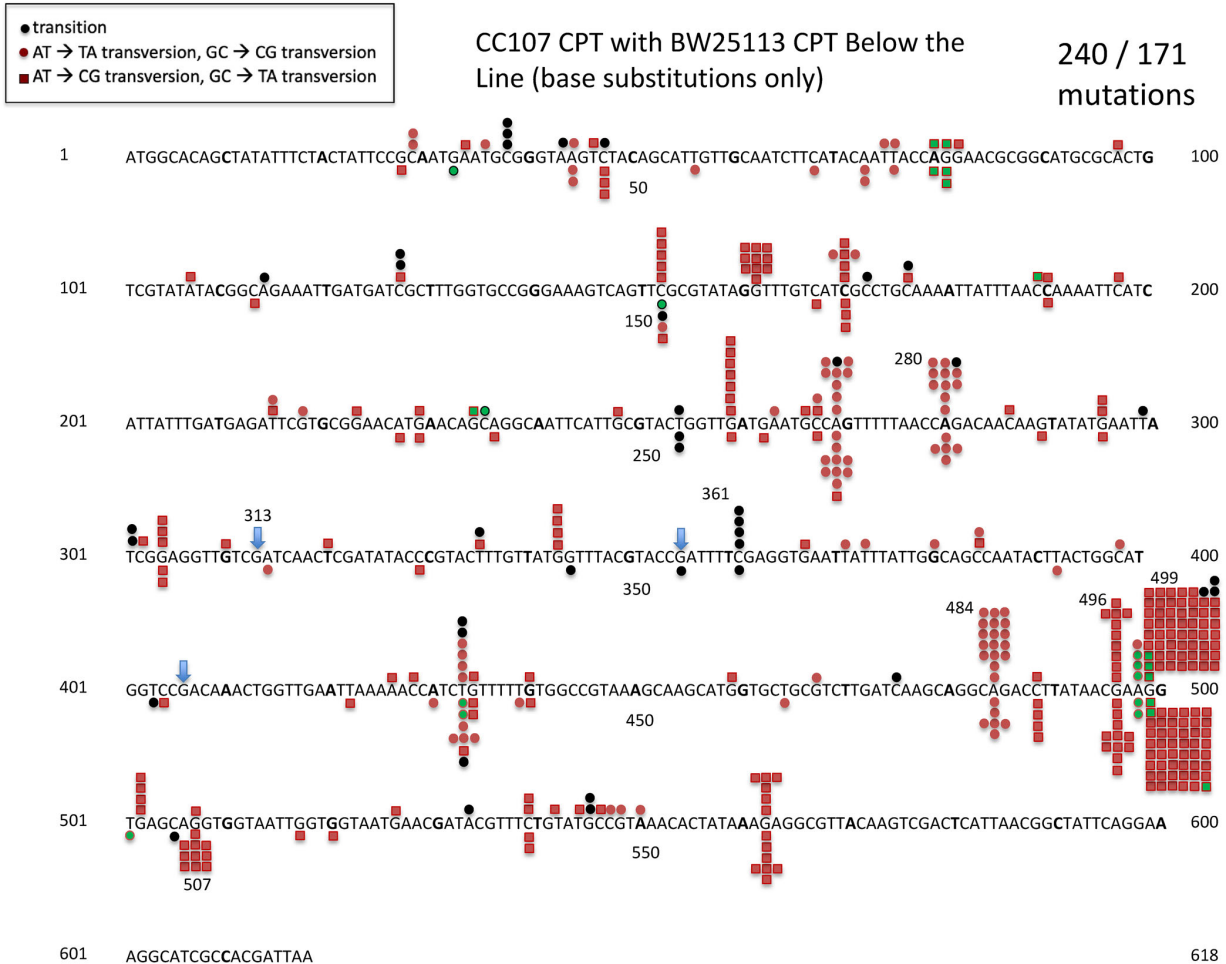
Distribution of CPT‐induced base substitution mutations in the *tdk* gene in CC107 and BW25113. Tandem double mutations are indicated by neighboring green squares.

We mutagenized each of the two strains with CPT. Table 1 shows the AZT‐resistant mutant frequencies, revealing a 13–30‐fold increase over the background, and Table 2 shows the distribution of mutations by type. Base substitutions predominate in the observed spectra, with the majority being either G:C →T:A or A:T →T:A changes, and about 10% being G:C→A:T changes (Table 3). Also, close to 1% of the mutations are tandem double mutations (data not shown, but see Figure 2). Figure 2 reveals the position and type of each base substitution in a total sample of 411 base substitution mutations. While Figure 2 allows one to see the spatial distribution of all the mutations, Tables 4 and 5 display the number of occurrences at each site for the two most prominent base substitutions, with the sites having the same nearest neighbors being grouped together. Here, the “triplet” involving the nearest neighbor on each side of the mutated base is shown in the 5′ to 3′ direction on the non‐transcribed strand, followed by the triplet on the opposing strand, also in the 5′ to 3; direction. For instance, the designation AGG/CCT 499 in Table 4 signifies that the G to T change at bp 499 occurs at the middle G of the AGG triplet, which on the other strand would be read CCT in the 5′ to 3′ direction. If a G to T change occurred at the middle G of an AGG triplet on the transcribed strand, then the designation for that site would be CCT/AGG. Tables 4 and 5 allow one to compare the number of occurrences in the samples at each site where the indicated base change is known to generate a mutant that is resistant to the selective agent AZT. In some cases no occurrences were detected after CPT mutagenesis, although examination of the mutations induced by other mutagens or mutators did detect mutants after the indicated change at those sites, or, the indicated change would result in a chain‐terminating nonsense mutation (data not shown). Thus, we can attach meaning to zero occurrences at certain sites.

**TABLE 1:** Mutant frequencies (*f*) of *Escherichia coli* using different strains and treatments.

Strain/treatment	Condition	Number of cultures	Frequency *f* (10^−8^)^a^
CC107 Spont	‐	12	92 (80–168)
CC107 CPT	100 μg/mL	7	2840 (2360‐3300)
BW25113 Spont	‐	17	153 (102–188)
BW25113 CPT 20 min	100 μg/mL	6	2040 (1170‐3280)
BW25113 CPT 40 min	100 μg/mL	6	2815 (1980‐2960)
BW25113 CPT 60 min	100 μg/mL	6	2280 (1690‐2550)
*uvrA* Spont	‐	28	111 (97–209)
*uvrA* CPT 20 min	50 μg/mL	6	626 (382–946)
*uvrA* CPT 30 min	50 μg/mL	6	605 (534–702)

^a^ Values in parenthesis are 95% confidence limits.

**TABLE 2:** Summary of mutations of *Escherichia coli* using different strains and treatments.

Name	BP	IS	In/Dels	Totals
CC107	26	7	8	41
CC107 CPT	240	24	42	306
BW25113	12	13	17	42
BW25113 CPT	171	2	20	193
*uvrA*	8	18	9	35
*uvrA* CPT	116	53	21	190

**TABLE 3:** Single base pair changes by strain.

Base change	CC107 CPT	BW25113 CPT	*uvrA* CPT	Total
GC→TA	135	106	61	302
AT→TA	49	35	28	112
GC→AT	20	4	14	38
AT→CG	9	5	5	19
AT→GC	10	4	4	18
GC→CG	12	2	3	17
Total	235	156	115	506

**TABLE 4:** G:C→T:A mutations induced by CPT.

	Position (bp)	Triplet	CC107	BW25113	Total (CC107 and BW25113)	*uvrA*
	499	AGG/CCT	55	46	101	19
	507	AGG/CCT	1	10	11	13
	488	CCT/AGG	1	4	5	2
	33	TGA/TCA	0	0	0	1
	211	TGA/TCA	0	0	0	0
	68	TGA/TCA	0	0	0	0
	229	TGA/TCA	1	1	2	1
	259	TGA/TCA	7	1	8	0
	262	TGA/TCA	0	1	1	0
	295	TGA/TCA	2	1	3	0
	367	TGA/TCA	1	0	1	0
	418	TGA/TCA	0	0	0	0
	502	TGA/TCA	4	0	4	1
	526	TGA/TCA	1	0	1	0
	167	TCA/TGA	0	1	1	0
	197	TCA/TGA	1	0	1	0
	200	TCA/TGA	0	0	0	0
	343	TGG/CCA	4	0	4	1
	442	TGG/CCA	0	0	0	2
	459	TGG/CCA	1	0	1	0
	517	TGG/CCA	0	1	1	1
	520	TGG/CCA	0	1	1	0
	190	CCA/TGG	1	1	2	0
	289	CCA/TGG	0	1	1	0
	127	TCG/CGA	1	0	1	1
	152	TCG/CGA	5	1	6	0
	170	TCG/CGA	4	3	7	1
	302	TCG/CGA	1	0	1	0
	496	CGA/TCG	10	11	21	2
	313	CGA/TCG	0	0	0	0
	355	CGA/TCG	0	0	0	0
	406	CGA/TCG	0	0	0	0
	113	GCA/TGC	0	1	1	0
	176	GCA/TGC	1	0	1	1
	398	GCA/TGC	0	0	0	0
	452	GCA/TGC	0	0	0	2
	456	GCA/TGC	0	0	0	0
	37	TGC/GCA	0	0	0	1
	248	TGC/GCA	1	0	1	0
	266	TGC/GCA	1	0	1	0
	545	TGC/GCA	0	0	0	0
	310	TGT/ACA	1	0	1	0
	434	TGT/ACA	1	2	3	1
	440	TGT/ACA	1	1	2	0
	541	TGT/ACA	0	0	0	0
	286	ACA/TGT	1	0	1	0
	41	GGT/ACC	0	0	0	0
	161	GGT/ACC	10	0	10	1
	344	GGT/ACC	0	0	0	0
	518	GGT/ACC	0	0	0	0
	428	ACC/GGT	1	0	1	0
	82	GGA/TCC	1	0	1	0
	223	GGA/TCC	1	0	1	1
	304	GGA/TCC	3	2	5	1
	598	GGA/TCC	0	0	0	0
	26	TCC/GGA	0	0	0	0
	404	TCC/GGA	0	1	1	0
	65	AGA/TCT	0	0	0	0
	115	AGA/TCT	0	0	0	0
	562	AGA/TCT	6	7	13	5
	47	TCT/AGA	0	3	3	0
	539	TCT/AGA	2	2	4	0
	504	AGC/GCT	0	0	0	0
	267	GCC/GGC	1	1	2	0
	384	GCC/GGC	1	0	1	0
	546	GCC/GGC	1	0	1	0
	380	GGC/GCC	0	0	0	0
	443	GGC/GCC	0	0	0	0
	45	AGT/ACT	0	0	0	1
	390	ACT/AGT	0	0	0	0
	534	ACG/CGT	0	0	0	0
	329	CCC/GGG	0	1	1	2
	38	GCG/CGC	0	0	0	0
	154	GCG/CGC	0	0	0	0
	249	GCG/CGC	0	0	0	0
	466	GCG/CGC	0	0	0	0
	128	CGC/GCG	0	0	0	0
	28	CGC/GCG	0	1	1	0
Total	78		135	106	241	61

**TABLE 5:** A:T→T:A mutations induced by CPT.

	Position (bp)	Triplet	CC107	BW25113	Total (CC107 and BW25113)	*uvrA*
	269	CAG/CTG	8	9	17	2
	280	CAG/CTG	10	5	15	7
	484	CAG/CTG	17	6	23	7
	433	CTG/CAG	4	4	8	3
	464	CTG/CAG	0	1	1	0
	15	ATT/AAT	0	0	0	0
	24	ATT/AAT	0	0	0	0
	75	ATT/AAT	1	0	1	0
	215	ATT/AAT	1	0	1	0
	373	ATT/AAT	1	0	1	0
	515	ATT/AAT	0	0	0	1
	76	TTA/TAA	1	1	2	0
	182	TTA/TAA	0	0	0	0
	203	TTA/TAA	0	0	0	0
	275	TTA/TAA	0	0	0	0
	299	TTA/TAA	0	0	0	0
	347	TTA/TAA	0	0	0	0
	371	TTA/TAA	1	0	1	0
	392	TTA/TAA	0	1	1	0
	422	TTA/TAA	0	0	0	0
	569	TTA/TAA	0	0	0	0
	584	TTA/TAA	0	0	0	0
	43	TAA/TTA	0	0	0	0
	448	TAA/TTA	0	0	0	0
	550	TAA/TTA	1	0	1	0
	559	TAA/TTA	0	0	0	0
	44	AAG/CTT	1	2	3	3
	498	AAG/CTT	1	0	1	0
	56	TTG/CAA	0	1	1	1
	59	TTG/CAA	0	0	0	1
	164	TTG/CAA	0	0	0	0
	439	TTG/CAA	0	1	1	0
	73	CAA/TTG	0	2	2	0
	36	ATG/CAT	1	0	1	0
	92	ATG/CAT	0	0	0	1
	342	ATG/CAT	0	0	0	0
	430	CAT/ATG	0	1	1	0
	251	GTA/TAC	0	0	0	0
	332	GTA/TAC	0	0	0	0
	348	TAC/GTA	0	0	0	0
	441	GTG/CAC	0	0	0	1
	142	GAA/TTC	0	0	0	0
	263	GAA/TTC	1	0	1	0
	159	TAG/CTA	0	0	0	1
	409	TCA/TGA	0	0	0	0
	108	ATA/TAT	0	0	0	0
	492	ATA/TAT	0	0	0	0
	558	ATA/TAT	0	0	0	0
	294	ATG/CAT	0	0	0	0
	314	GAT/ATC	0	1	1	0
	339	GTT/AAC	0	0	0	0
	435	GTT/AAC	0	0	0	0
	178	AAA/TTT	0	0	0	0
	424	AAA/TTT	0	0	0	0
Total	55		49	35	84	28

It is evident from Figure 2 that there is a major hotspot at position 499, which has 102 of the 411 base changes (24%). Note that this site involves a G:C→T:A change at an AGG triplet, one of the favored triplets based on previous work in mammalian cell lines (e.g., de Boer & Glickman, 1989). In fact, the region from 484 to 507 has all the earmarks of an mutation prone region (MPR; Mashiach et al., 2021), with 174 of the 411 base substitution mutations (42%) in a 23‐base pair region. This involves 9 different sites, including another AGG site. There are still other AGG triplets in the tdk gene at positions 81 and 161 that do not show mutations, but a G:C→T:A change at these latter two sites very probably does not yield a mutant phenotype. Thus, we cannot say that these sites are “cold” because they are outside of an MPR region. However, mutations at the triplet CGA do allow us to distinguish between regions. There are four CGA triplets at which we have found G:C→T:A mutations that yield AZT‐resistant mutants, at positions 313, 355, 406, and 496. The number of occurrences at each of these sites in the total CPT treated sets are, respectively, 0, 0, 0, and 21 (*p* = 4^21^/4 = 4^20^; *p* < 10^−12^). (The sites at 313, 355, and 406 are indicated by short blue arrows in Figure 2.) Thus, the site in the MPR, 496, is hot, and the other sites that are outside this region are “cold.” We do not have enough data at this point to define other MPRs, but the region from 259 to 280 might turn out to be one with additional work.

The CAG triplet is clearly a favored site for the A:T→T:A change. This site seems to be well induced even if on the transcribed strand (note that CTG on the non‐transcribed strand is the complement of CAG on the transcribed strand). Almost all of the well‐induced sites conform to the established rule of being at the 5′ purine of a purine‐purine sequence. The one prominent exception is at position 161, where it is the 3′ purine that is mutated.

If we want to define the region from 484 to 507 as a hot region (MPR), then we need to account for the relatively small number of occurrences at the AGG at 507, where we have a total of 11 occurrences, versus 107 at the very close AGG at 499. One possibility is that the UvrA,B,C excision repair system might be preferentially repairing lesions at 507 (see below).

### 3.3 CPT‐induced mutations in a UvrA‐deficient strain

**FIGURE 3:**
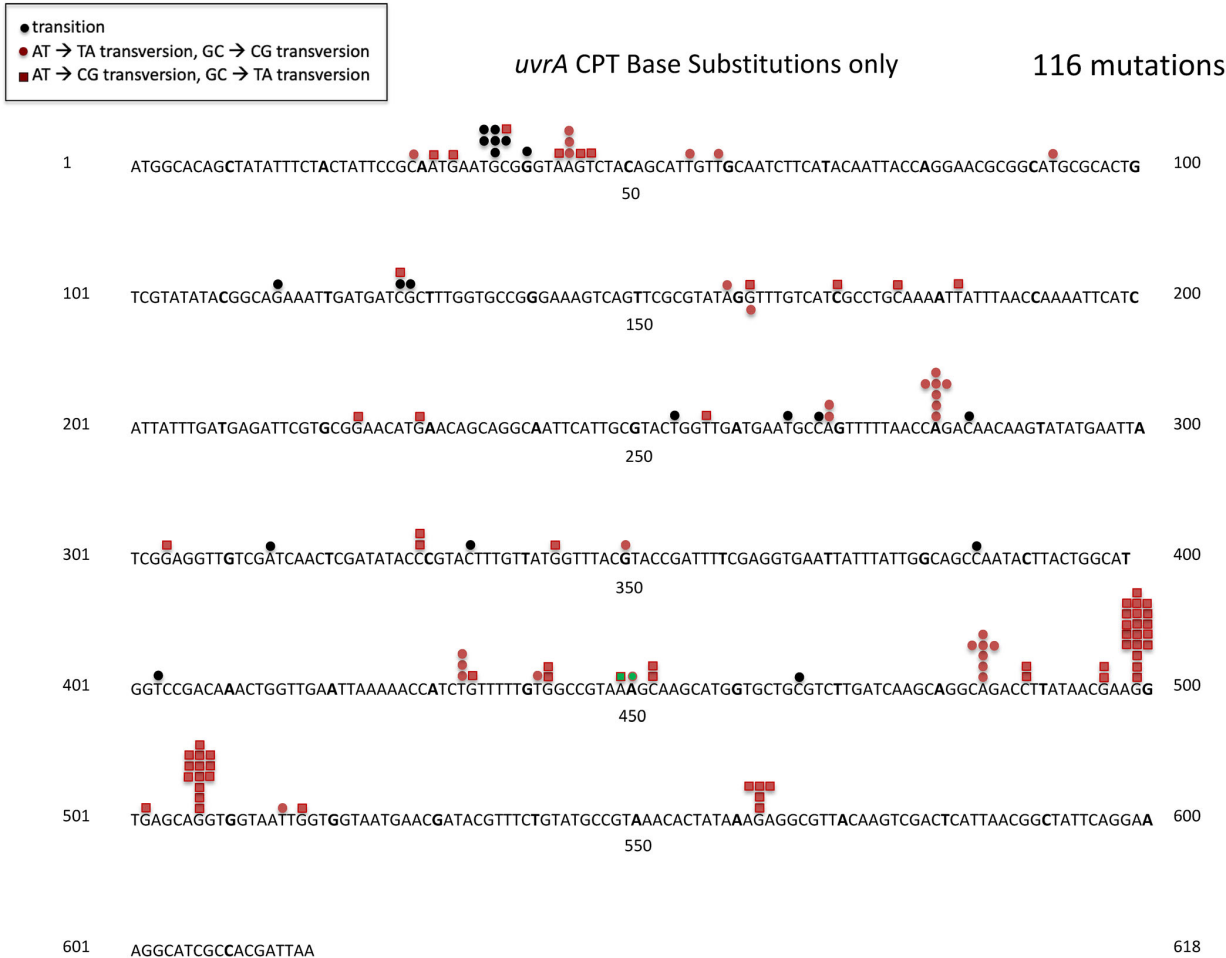
Distribution of CPT‐induced mutations in the *tdk* gene in a *uvrA* derivative of BW25113.

**FIGURE 4:**
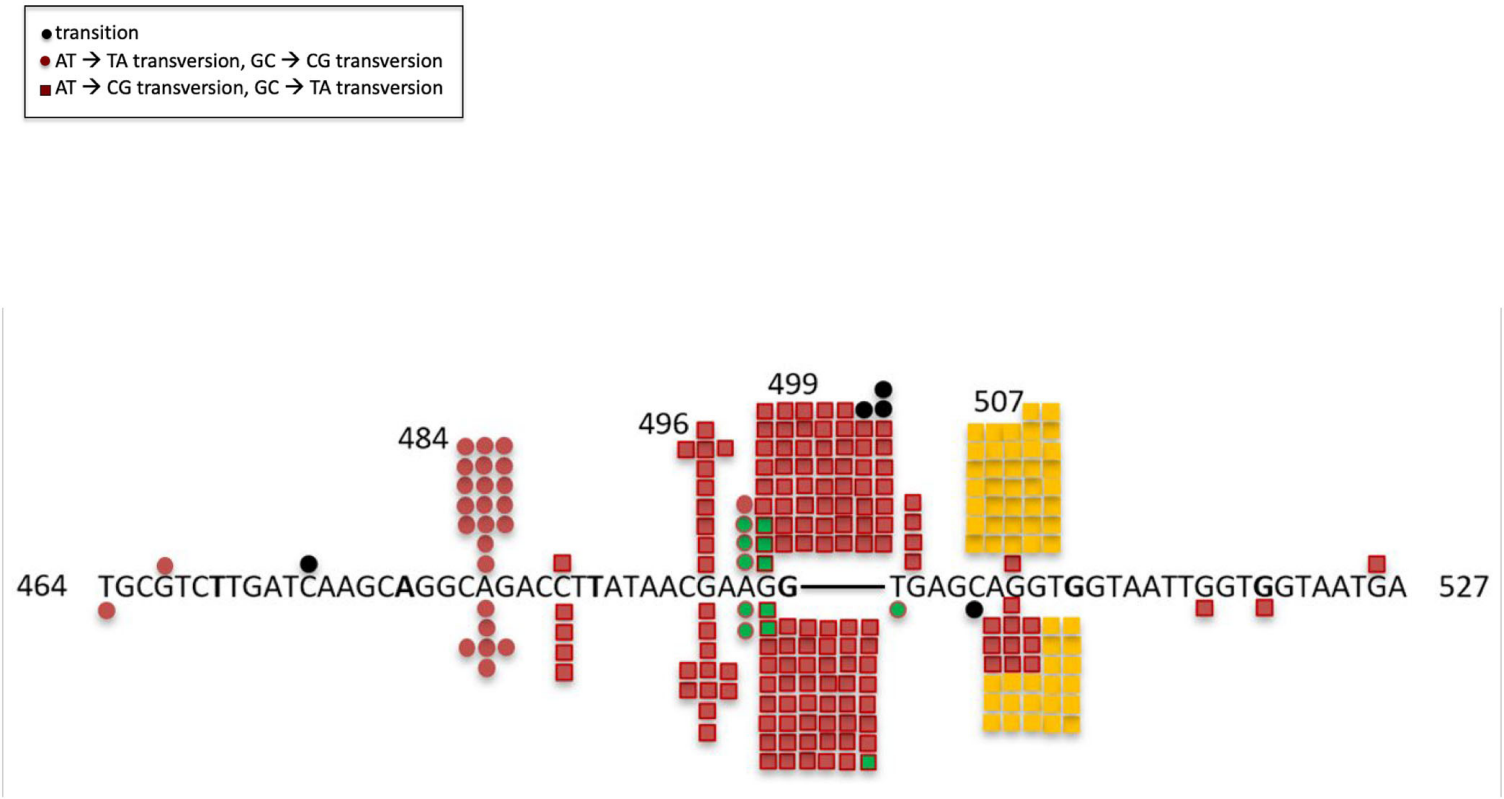
Distribution of CPT‐induced mutations in the segment of the *tdk* gene from bp 464–527 in CC107 and BW25113. Here one has extrapolated the expected number ofmutations occurring at position 507 if the same large number of mutations were analyzed in a *uvrA* derivative of each strain.

We mutagenized a strain lacking the *uvrA,B,C*‐encoded excision repair system, using a strain from the KEIO collection (see “Materials and Methods”) with a deletion of the *uvrA* gene. CPT pre‐mutational lesions can be removed by this system, although it has a strong preference for repairing lesions on the transcribed strand (“transcription‐coupled repair”; Kunala & Brash, 1992; Oller et al., 1992; see review by Selby et al., 2023). In one case a new hotspot for UV‐induced mutations does appear in the spectrum of *lacI* nonsense mutations in an excision repair‐deficient strain, arising from a lesion on the transcribed strand (Todd & Glickman, 1982). Mutagenizing a CPT‐sensitive strain such as *uvrA* with CPT causes extensive killing, and it is difficult to recover mutants, as one needs very precise conditions. In fact, one study even concluded that CPT cannot mutagenize an excision repair deficient strain (Brouwer et al., 1981). Nevertheless, we have been able to recover mutants after CPT treatment of a *uvrA* strain (see Tables 1, 2, 3). Figure 3 shows the distribution of 116 base substitution mutations. Here, we see at this stage of resolution two prominent hotspots, the G:C→T:A change at 499, as before, and now, the G:C→T:A change at 507. These two sites, with 19 and 13 occurrences, respectively, are essentially equivalent (*p* >.25; Chi‐square). Even taking the ratios of 19:13 of the occurrences at these two sites strictly as they are, we can extrapolate the number of occurrences expected in the wild‐type strains (Figure 2) if we multiply the detected occurrences at 499 by 13/19 (0.68). Figure 4 shows this, with the extrapolated occurrences shown in yellow. Thus, the site at 507 is preferentially repaired by the excision repair system, even though the lesion is on the non‐transcribed strand, effectively camouflaging this hotspot.

## 4 DISCUSSION: HOTSPOT ANALYSIS

One of the fascinating aspects of mutational hotspots is that despite the enormous amount of information gathered (Fernandez et al., 2020, and references therein), there are still important unanswered questions. The field has uncovered much about the sequence preferences of different mutagens and mutational processes. In many cases, nearest neighbors play a role in determining preferential sites (Coulondre & Miller, 1977; Fernandez et al., 2020; Foster et al., 2018; Miller, 1985; Pienkowska et al., 1993), and this has been used as the basis for “mutational signatures” in cultured mammalian cells as revealed by genomic sequencing (e.g., Phillips, 2018), However, there are significant differences in mutation rates for specific mutagens that approach two orders of magnitude, even for the same base change among sites with the same nearest neighbors (e.g., Miller, 1985). There are also large differences between sites with both the same first and second nearest neighbors on each side of the mutated base (Miller, 1985). As powerful as genomic sequencing is, it cannot distinguish different mutation rates between specific sites with the same nearest neighbors, as one needs to analyze the different rates at these sites by examining repeated occurrences. We recently analyzed a large sample of mutations in the *thyA* gene of *E. coli* induced by different mutagens and mutator genes and provided evidence that there are certain mutation‐prone regions (MPRs) of genes that are inter‐dispersed between regions that are much less mutation‐prone (Mashiach et al., 2021), the differences varying between 3‐ and8‐fold. In that study, we used 10 different mutagenesis conditions (2AP [2‐aminopurine], EMS [ethyl methanesulfonate], etc.), and sequenced over 1000 mutations. The aim was to have biases cancel out by the large variety of conditions. However, it is important to supplement that study not only with the same analysis applied to a second gene but also with the in‐depth examination of mutations induced by a single treatment, where one can analyze more easily the distribution of mutations at sites with identical nearest neighbors. Therefore, in the work reported here we sought to examine the hotspots for the mutagen CPT in a different target, the *tdk* gene in *E. coli*. (In a subsequent study we will repeat the Mashiach et al. analysis in the *tdk* gene.) We also demonstrate the utility of the *tdk* reporter gene system.

CPT has preferred nearest neighbors. In *E. coli*, The most favored triplets include AGG and CGA for the G:C to AT change, and CAG for the A:T to T:A change. However, other triplets such as TGG are also somewhat favored. In the work reported in *thyA*, we did not find hotspots at the one AGG triplet, at any of the four CGA triplets, the two CAG triplets, or at three of the four TGG triplets. None of these triplets are located in any of the three MPRs that we defined. Here, we are looking at the triplets that have the CPT target on the non‐transcribed strand, as the *uvrA.B,C* encoded excision repair system preferentially repairs CPT lesions on the transcribed strand via “transcription coupled repair” (Kunala & Brash, 1992; Oller et al., 1992; Selby et al., 2023). The only hotspot in *thyA* is at one of the three TGG triplets that is in fact at one of the MPRs. Although these findings are nicely explained by the MPR idea, the one hotspot is in the only region for which one has shown that the secondary structure plays a role in a spontaneous hotspot, albeit via a novel mechanism not involving chemically induced lesions. We therefore sought to use a different gene system to examine hotspots among CPT‐induced mutational sites.

The tdk/AZT‐resistant system we developed here is an ideal gene reporter for mutations. The *tdk* gene is only 618 bp long, meaning that one can sequence the whole gene easily with a single primer pair. AZT‐resistant mutants appear on LB plates containing 100 ng/mL AZT. This is an advantage because here mutants appear after 1 day, whereas in some reporter systems, such as the *thyA*/trimethoprim resistance system, it takes 2 days for full mutant colonies to appear. Also, any mutation resulting in an inactive gene product, including base substitutions, insertions, and deletions will show up in this system. Another advantage is the low background of spontaneous base substitutions. The spontaneous mutations consist of 20%–25% IS insertions, and the spontaneous base substitutions do not have evident hotspots that can interfere with the analysis of induced mutations. Also, the *tdk* gene is more AT‐rich than most genes in *E. coli*, offering an interesting comparison with other reporter gene results. The analysis of 411 CPT‐induced mutations in *tdk* seen in Figure 2 shows a major hotspot at position 499 comprising 25% of all the base pair substitution mutations (see also Table 4). In fact, the region between bp 484 and 507 has all the hallmarks of a mutation prone region, with a number of sites that are well induced. The major hotspot at 499 is at the AGG codon. At first glance, the relatively low number of occurrences at the AGG triplet at bp 507 would appear to contradict the idea that the reason the AGG triplet is particularly hot at 499 is that it is in an MPR, as 507 is so close. However, when we analyzed CPT‐induced mutations in a *uvrA* excision repair deficient strain (Figure 3), now the site at 507 also appears as a hotspot at nearly the same level as the site at 499. This result shows that even though we are looking at mutations that occur on the non‐transcribed strand, as that is where the adjacent purines are, still the UvrABC excision repair system preferentially removes premutational lesions at certain positions, in this case at 507. If we take the ratio of occurrences at 507 versus occurrences at 499 from Figure 3, we can extrapolate from the data in the wild‐type strains (Figure 2) to estimate the true number of occurrences at 507 that we would see. These are shown in yellow color in Figure 4, to give a more accurate picture of the hotspot at 507 when we remove the “camouflage” of the preferential repair of lesions at 507.

While it is clear that the two AGG triplets at 499 and 507 represent major hotspots, we cannot say that the other AGG triplets, at positions 61, 108, and 527 are not hotspots, since mutations here may not result in an AZT‐resistant phenotype. (The AGG triplet on the other strand is only sparsely represented.) In the wild‐type strain, this can be explained by the UvrABC excision repair system acting preferentially on the transcribed strand. The failure to see a real hotspot here in the UvrA‐deficient strain might be because it is only at the boundary of the mutationally prone region. Alternatively, it could represent again a preference of the excision repair system itself for certain proximal surrounding regions. However, we can compare the four CGA triplets in the gene at 313, 355, 406, and 494. We have detected G:C→T:A mutations at each of these sites in the spectra of other mutagens or mutators (data not shown). As can be seen in Table 3 and Figure 3, the number of occurrences at 313, 355, 406, and 494, respectively, in the combined samples of the CPT‐induced mutations (Figure 3) are 0, 0, 0, and 21 (*p* = < 10^−12^). Therefore, when the triplet CGA appears within an MPR, as 494 does, it generates a hotspot, otherwise it does not.

## 5 CONCLUSIONS

The elements necessary for a significant hotspot using CPT as an example are:

1. Preferential lesion target and type of mutation, for example, G: C→T:A, and A:T→T:A at the 5′‐end of a pur–pur sequence.
2. Preferential nearest neighbors in many cases.
3. Preferential location in a gene, namely in an MPR.
4. To observe the full complement of hotspots one needs to unmask the effect of repair systems, particularly the UvrA,B,C excision repair systems, as they can preferentially remove lesions, camouflaging certain hotspots.

## AUTHOR CONTRIBUTIONS

Jeffrey H. Miller designed the study. Courtney Young, Mackenzie Lee, Zoe Ge, Jeana Shin, Bella Bursulaya, Dana Sorensen, Arnav Saud, Ananya Sridharan, Ava Gonick, Nhu Phi, Kelly Nguyen, Shawal Bhalli, and Jyotsna Hiranandani performed the experiments and generated the figures. Jeffrey H. Miller wrote the manuscript. All authors reviewed the submitted version of the manuscript.

## FUNDING INFORMATION

This work was partially funded by a Faculty Research Grant from the University of California.

## CONFLICT OF INTEREST STATEMENT

The authors declare no competing interests.

## Data Availability

The data that support the findings of this study are available on request from the corresponding author. The data are not publicly available due to privacy or ethical restrictions.
